# First-pass contrast-enhanced imaging versus equilibrium-phase T1 mapping for determining the distribution volume of gadolinium

**DOI:** 10.1186/1532-429X-13-S1-P17

**Published:** 2011-02-02

**Authors:** Michael Salerno, Patrick Antkowiak, John J Green, Rajesh Janardhanan, Ronny A Jiji, Christopher M Kramer, Frederick H Epstein

**Affiliations:** 1 University of Virginia, Charlottesville, VA, USA

## Introduction

Late gadolinium enhancement can identify focal fibrosis, but cannot evaluate diffuse myocardial fibrosis. Multiple methods have been used to determine the partition coefficient (λ) and volume of distribution (Vd) of gadolinium to quantify diffuse myocardial fibrosis, including first-pass and T1 mapping based techniques. These two techniques have not been directly compared in the same subjects.

## Purpose

To directly compare the myocardial Vd in normal subjects as determined from first-pass contrast-enhanced imaging with modified Kety analysis versus T1 mapping at contrast equilibrium following continuous infusion of Gd.

## Methods

First-pass contrast-enhanced imaging and T1 mapping measurements were performed in 5 healthy volunteers (age 36±11) on a Siemens 1.5T Avanto. Hematocrit was measured to accurately determine Vd from λ. First pass measurements were performed during a bolus injection of 0.1mmol/kg Gd-DTPA with a dual-contrast saturation recovery GRE-EPI pulse sequence. Sequence parameters included: TE/TR/ETL/FA1.1ms/6.1ms/4/25°, 270x340mm FOV, resolution 2.7x2.7mm, thickness 10mm. First-pass data was analyzed using a modified Kety model which enabled determination of Vd as well as Ktrans to quantify myocardial perfusion. T1 maps were determined using a MOLLI pulse sequence pre-contrast and during continuous infusion of 0.001 mmol/kg Gd until equilibrium was achieved. Sequence parameters included: TE/TR/FA 1.1 ms,/2.5ms/35°, FOV= 340 x 260, resolution 1.8mm x 1.8mm, thickness 8mm. The λ was determined as the slope of the linear fit of the data on a plot of 1/T1 myocardium versus 1/T1 blood. The Vd was calculated as λ*(1-Hct). Processing of all data was performed with in-house MATLAB programs.

## Results

The mean Ktrans determined from first-pass data was 0.41±0.02. The mean Vd values, while similar, were higher from the first pass technique. (0.34±0.05 for first-pass versus 0.29±0.01 for T1 mapping p=0.02).

## Conclusions

First-pass contrast-enhanced and T1 mapping techniques provide similar measures of Vd of gadolinium in normal subjects, yet each has specific advantages. First-pass imaging is rapid, but spatial resolution and SNR are limited. Equilibrium T1 mapping with continuous infusion is less time-efficient, but spatial resolution and SNR are improved.

